# A Novel Hybrid Peptide VLP-Aβ Exhibits Antioxidant Activity In Vitro and In Vivo via KEAP1-NRF2-ARE Signaling Pathway

**DOI:** 10.3390/antiox14050583

**Published:** 2025-05-12

**Authors:** Junyong Wang, Wenxiu Zhang, Rijun Zhang, Xuelian Zhao, Jing Zhang, Yichen Zhou, Yucui Tong, Zaheer Abbas, Zhenzhen Li, Haosen Zhang, Di Yang, Sichao Chen, Cong Hu, Dayong Si, Xubiao Wei

**Affiliations:** 1 Laboratory of Feed Biotechnology, State Key Laboratory of Animal Nutrition and Feeding, College of Animal Science and Technology, China Agricultural University, Beijing 100193, China; wangjy9722@cau.edu.cn (J.W.);; 2 Beijing Dabeinong Technology Group Co., Ltd., Beijing 100194, China

**Keywords:** hybrid peptide, oxidative stress, KEAP1-NRF2, hydrogen peroxide, liver damage

## Abstract

Oxidative stress plays a crucial role in the development and progression of various diseases. Antioxidant peptides have attracted great attention in agricultural, food, and clinical fields due to their low toxicity, high efficacy, and easy absorption, but the development of antioxidant peptides and their in-depth molecular mechanisms are still lacking. The previous study established a platform for the high-throughput design and screening of multifunctional peptides and successfully identified a novel hybrid peptide, VLP-Aβ (VA), which exhibits both antioxidant and immunomodulatory properties. This study aimed to evaluate the antioxidant activity of VA and investigate the underlying molecular mechanisms. The antioxidant effects of VA were evaluated using both in vitro (H_2_O_2_-induced oxidative damage in HepG2 cells) and in vivo (CCl_4_-induced liver damage in mice) models. VA exhibited significant antioxidant activity both in vitro and in vivo, significantly improving the cell viability and increasing the levels of antioxidant enzymes (SOD, CAT, GSH-Px) to alleviate oxidative stress. These findings indicated that the antioxidant effect of VA is dependent on NRF2, as evidenced by NRF2 knockdown experiments. Further investigation revealed that VA alleviates oxidative stress by modulating the KEAP1-NRF2-ARE signaling pathway. These findings provide insights into the properties of the antioxidant peptide VA, expand the understanding of its molecular mechanisms, and suggest new opportunities for developing VA as a novel functional agent in the agricultural, food, and clinical industries.

## 1. Introduction

Redox reactions are critical in many physiological processes in the body, as humans and animals are frequently exposed to high levels of oxidative and electrophilic chemicals [1,2,3,4]. Under normal physiological conditions, the body maintains a balance in the redox system. However, once this balance is disrupted, it leads to the accumulation of reactive and electrophilic substances, resulting in oxidative stress. The primary contributors to oxidative stress are excess reactive oxygen species (ROS), including superoxide anion radicals (O_2_^•−^), hydrogen peroxide (H_2_O_2_), and hydroxyl radicals (^•^OH) [5,6,7]. The excessive accumulation of these free radicals can damage cellular DNA, proteins, and lipids, leading to cellular dysfunction and a range of diseases including cancer, diabetes, hypertension, and cystic fibrosis [6,8,9]. Given the critical role of oxidative stress in these diseases, there is a growing need for effective antioxidant agents, such as antioxidant peptides, to mitigate these detrimental effects.

In recent years, antioxidant peptides have attracted increasing attention in agricultural, food, and clinical fields due to their low toxicity, high efficacy, and easy absorption [10,11,12]. However, the development of antioxidant peptides still faces great challenges, and the limited functionality of peptides is a major barrier to their application. Heterogeneous design is an efficient and simple peptide design method that combines peptide fragments with different functionalities to create novel multifunctional bioactive peptides. Previous studies have synthesized and screened hybrid peptides with both immune regulatory and antioxidant activities, and among them, the hybrid peptide, VA, exhibited particularly significant activity (Table 1) [13].

The hybrid peptide, VA, is a dual-functional bioactive peptide composed of the parental peptides, VLP and Aβ. VLP is a bioactive peptide derived from casein hydrolysate isolated from buffalo milk [14]. VLP possesses a wide range of functional activities, including antioxidant effects [15,16], antiosteopenic effects [17], antiaging effects [18], and enhanced calcium transport and absorption [19]. On the other hand, amyloid β-protein (Aβ) is a biomolecule closely associated with Alzheimer’s disease. Under pathological conditions, Aβ aggregates, transitioning from monomers to oligomers and fibrils, ultimately forming amyloid plaques. These plaques lead to strong neurotoxicity and harm to the body. The negative effects of Aβ are primarily attributed to the oligomeric form; however, there are few reports on the effects of Aβ monomers [20]. Previous studies have shown that Aβ monomers have a strong immune-activating capability, which is why they were used as immune-regulatory parental peptides in the hybrid design [13]. This study aims to explore the antioxidant function of the hybrid peptide, VA, and its potential underlying mechanisms through in vitro and in vivo experiments.

## 2. Materials and Methods

### 2.1. Chemicals and Reagents

The peptides Aβ, VLP, and VA were synthesized by GL Biochem Ltd. (Shanghai, China) with a purity of 95%. All the peptide stock solutions were fully dissolved in DMSO and diluted to the desired working concentrations as required during the experimental procedures. The HepG2 cell line was obtained from the Shanghai Cell Bank, Institute of Cell Biology, Chinese Academy of Sciences (Shanghai, China). Dulbecco’s Modified Eagle’s Medium (DMEM) was purchased from Gibco (Waltham, MA, USA). Fetal bovine serum (FBS) was obtained from Procell (Wuhan, China). Penicillin-streptomycin, RIPA buffer, DCFH-DA, mitochondrial membrane potential assay kit with JC-1 (cat. no. M8650), CCK-8 (cat. no. CA1210), BCA (cat. no. PC0020), SOD (cat. no. BC5165), CAT (cat. no. BC0205), GSH-Px (cat. no. BC1195), MDA (cat. no. BC0025), AST (cat. no. BC1565), and ALT (cat. no. BC1555) assay kits were purchased from Beijing Solarbio Science & Technology Co., Ltd. (Beijing, China). The terminal deoxynucleotidyl transferase-mediated dUTP nick-end labeling (TUNEL) assay kit was purchased from Servicebio (Wuhan, China). Hydrogen peroxide (H_2_O_2_) and silymarin were purchased from Sigma-Aldrich (St. Louis, MO, USA). Lipo8000™ reagent and ECL were purchased from Beyotime Biotechnology (Shanghai, China). siRNA targeting NRF2 and control siRNA were purchased from Sangon Biotech (Shanghai, China). TRIzol reagent, a reverse transcription kit, and SYBR mix were purchased from Vazyme (Nanjing, China). All other analytical grade reagents were obtained from China National Medicines Corporation Co., Ltd. (Beijing, China). The anti-NRF2 (cat. no. ab62352) and anti-HO-1 (cat. no. ab189491) antibodies were purchased from Abcam (Cambridge, UK). The anti-KEAP1 (cat. no. 10503-2-AP) and anti-β-actin (cat. no. 20536-1-AP) antibodies and secondary antibodies were purchased from Proteintech (Wuhan, China).

### 2.2. Cell Culture

HepG2 cells were cultured in DMEM supplemented with 10% FBS and 1% penicillin-streptomycin. The cells were incubated at 37 °C in a humidified atmosphere containing 5% CO_2_ and passaged every other day.

### 2.3. H_2_O_2_-Induced HepG2 Cell Damage Model

HepG2 cells were seeded at 2 × 10^5^ cells per well in 96-well plates and cultured overnight. The medium was then removed, and 100 μL of fresh DMEM was added to each well, followed by a 2 h incubation. H_2_O_2_ was subsequently added to achieve final concentrations of 450, 500, 550, 600, 650, 700, 750, 800, and 850 μM, and the cells were incubated for an additional 6 h. The cell viability was assessed using the CCK-8 kit according to the manufacturer’s instructions, and the IC_50_ of H_2_O_2_ on HepG2 cells was calculated.

### 2.4. Protective Effect of the Hybrid Peptide, VA, on H_2_O_2_-Induced Cell Damage

HepG2 cells were seeded at 2 × 10^5^ cells per well in 96-well plates and incubated overnight. After removing the culture medium, 100 ng/mL of peptides (VA, Aβ, or VLP) were added, and the cells were incubated for 2 h. H_2_O_2_ was subsequently added to achieve a final concentration of 650 μM, and the cells were incubated for an additional 6 h. The cell viability was assessed using the CCK-8 assay, with the model group receiving only H_2_O_2_ and the control group receiving only DMEM.

### 2.5. Detection of Cellular ROS Levels

HepG2 cells were seeded at 2 × 10^6^ cells per well in 12-well plates and incubated overnight. After removing the medium, 100 ng/mL of peptides (VA, Aβ, or VLP) were added, and the cells were incubated for 2 h. H_2_O_2_ was subsequently added to achieve a final concentration of 650 μM, and the cells were incubated for an additional 6 h. After incubation, the medium was replaced, and 10 μmol/L DCFH-DA was added to the cells for a 30 min incubation in the dark. The cells were washed twice with PBS, and the ROS fluorescence was observed using a fluorescence microscope. For fluorescence intensity quantification, after incubation with H_2_O_2_, the cells were digested using trypsin and washed with PBS, then incubated with DCFH-DA for 30 min in the dark. After washing twice with PBS, the cell was resuspended in 1 mL of PBS, and 200 μL of the suspension was transferred to a 96-well plate for detection using a multifunctional microplate reader.

### 2.6. Detection of Cellular Mitochondrial Damage

HepG2 cells were seeded at 2 × 10^6^ cells per well in 12-well plates and incubated overnight. After removing the medium, different concentrations of the VA peptide (100, 200, and 500 ng/mL) were added, and the cells were incubated for 2 h. H_2_O_2_ was subsequently added to achieve a final concentration of 650 μM, and the cells were incubated for an additional 6 h. After incubation, the medium was replaced with 500 μL of 1× JC-1 staining solution and incubated in the dark at 37 °C for 20 min. The cells were washed twice with 1× washing solution and observed using a fluorescence microscope.

### 2.7. Measurement of the Cellular Antioxidant Enzyme Activity

HepG2 cells were seeded at 4 × 10^6^ cells per well in 6-well plates and incubated overnight. After removing the culture medium, 100 ng/mL of peptides (VA, Aβ, or VLP) were added, and the cells were incubated for 2 h. H_2_O_2_ was subsequently added to achieve a final concentration of 650 μM, and the cells were incubated for an additional 6 h. After incubation, the cells were digested using trypsin, washed twice with PBS, and resuspended in 200 μL of protein extraction buffer. Protein extraction was performed using an ultrasonic homogenizer at 200 W for 3 s with a 10 s interval, repeated 30 times. The homogenate was centrifuged at 8000× *g* for 10 min at 4 °C, and the supernatant was collected. The protein concentration was determined using the BCA assay. The antioxidant enzyme activity (SOD, CAT, GSH-Px, MDA) was measured using the corresponding assay kits according to the manufacturer’s instructions.

### 2.8. Small Interfering RNA (siRNA) Transfection

The siRNA targeting the NRF2 sequence was as follows: si-NRF2 (sense): 5′-CAGUCUUCAUUGCUACUAATT-3′; si-NRF2 (antisense): 5′-UUAGUAGCAAUGAAGACUGTT-3′. HepG2 cells were transfected using Lipo8000™ reagent, according to the manufacturer’s instructions. Briefly, HepG2 cells were seeded in 6-well plates at a density of 4 × 10^6^ cells per well and incubated overnight. A transfection mixture containing 125 μL of Opti-MEM, 4 μL of Lipo8000, and 4 μL of si-NRF2 (100 pM) was prepared and added to the 6-well plates, followed by a 12 h incubation. After 12 h, the medium was replaced with the complete medium, and the cells were cultured for an additional two days. Subsequently, the cells were treated with hydrogen peroxide (H_2_O_2_) for subsequent analysis. The control group was transfected with an equivalent amount of negative control siRNA.

### 2.9. qRT-PCR

HepG2 cells were collected and washed three times with PBS after trypsin digestion. The RNA was extracted using TRIzol reagent. The RNA purity and concentration were assessed using a nanodrop spectrophotometer. cDNA was synthesized using a reverse transcription kit according to the manufacturer’s instructions. The primers used in this study are listed in Table 2. The PCR reaction system (20 μL total volume) contained 0.4 μL of forward primer (100 μM), 0.4 μL of reverse primer (10 μM), 2 μL of cDNA (2.5 ng/μL), 10 μL of 2× SYBR Mix, and 7.2 μL of ddH_2_O. PCR was performed using the following two-step amplification program: pre-denaturation at 95 °C for 30 s, denaturation at 95 °C for 5 s, and extension at 60 °C for 30 s for a total of 40 cycles; a single-cycle melting curve program was then performed with the following parameters: 95 °C for 5 s, 60 °C for 60 s, and 95 °C for 1 s. Gene expression was normalized to β-actin as a housekeeping gene, and the results were reported as the fold change in the gene expression relative to the control samples.

### 2.10. Western Blot

The cells were collected, washed three times with PBS, and lysed using RIPA buffer supplemented with a protease inhibitor cocktail. The protein concentration was determined using the BCA assay. Proteins were separated via SDS-PAGE and transferred onto a PVDF membrane. The membrane was blocked with 5% non-fat milk at room temperature for 1 h, followed by overnight incubation with the primary antibody. Membranes were washed three times with TBST, incubated with the secondary antibody at room temperature for 1 h, and visualized using ECL reagent. Protein bands were quantified using Image J (Version: 1.54i).

The primary antibody dilution ratios used in this study were as follows: anti-NRF2 (1:2000), anti-HMOX1 (1:2000), KEAP1 (1:20,000), and β-actin (1:10,000). The secondary antibody, HRP-Goat Anti-Rabbit IgG, was diluted at a ratio of 1:5000.

### 2.11. Animal Models

Male C57BL/6 SPF mice (6 weeks old) were purchased from GemPharmatech Co., Ltd. (Nanjing, China). and maintained in cages under specific pathogen-free (SPF) conditions. The mice had free access to food and water throughout the experiment. All the animal procedures were performed in accordance with the guidelines provided by the Institutional Animal Care and Use Committee of China Agricultural University.

The mice were randomly divided into seven treatment groups (six mice per group): control group, CCl_4_ group (2% CCl_4_ at 5 mL/kg body weight), CCl_4_ + silymarin group (20 mg/kg body weight), CCl_4_ + Aβ group (10 mg/kg body weight), CCl_4_ + VLP group (10 mg/kg body weight), CCl_4_ + VA group (5 mg/kg body weight), and CCl_4_ + VAH group (10 mg/kg body weight). The mice were allowed to acclimatize for 5 days prior to the experiment. From day 0 to day 7, each group was intraperitoneally injected with the corresponding drug or peptide, with the control and CCl_4_ groups receiving an equivalent volume of saline. After 7 days, CCl_4_ was injected intraperitoneally into all the groups except the control group, which received an equivalent volume of olive oil. The mice were sacrificed after 16 h of CCl_4_ treatment, and blood and liver tissues were collected.

#### 2.11.1. Serum Biochemical Analysis

After the blood collection, the serum was separated via centrifugation at 5000 rpm for 20 min at 4 °C. Liver function markers (AST and ALT) were measured using commercial assay kits, following the manufacturer’s instructions.

#### 2.11.2. Measurement of Antioxidant Enzyme Activity in the Serum and Liver

Liver tissue was sectioned into small pieces, followed by homogenization with 500 μL of tissue lysis buffer using a tissue homogenizer. The homogenate was centrifuged at 12,000 rpm for 20 min at 4 °C to obtain the supernatant. The protein concentration was determined using the BCA assay. The antioxidant enzyme activity (SOD, CAT, GSH-Px) and MDA levels in both the serum and liver tissue homogenates were measured using commercial kits according to the manufacturer’s instructions.

#### 2.11.3. Liver Tissue Histological Analysis

Liver tissues were fixed in 4% formalin and embedded in paraffin. Sections (5 μm) were stained with H&E and Masson’s trichrome for histological examination under a microscope.

The liver tissue apoptosis was assessed by using the TUNEL assay. Tissue sections were dewaxed and stained using a TUNEL apoptosis detection kit following the manufacturer’s instructions, then observed using a fluorescence microscope.

#### 2.11.4. Liver Tissue qRT-PCR Analysis

Liver tissue was homogenized using TRIzol reagent for RNA extraction, as previously described. The RNA purity and concentration were determined using a nanodrop spectrophotometer. cDNA synthesis was performed as described earlier, and qRT-PCR was performed using the primers listed in Table 2. The gene expression was normalized to β-actin, and the results were reported as the fold increase relative to the control samples.

### 2.12. Statistical Analysis

Data were analyzed using GraphPad Prism 9 software. All the results are presented as the mean ± standard error of the mean (SEM) from at least three independent experiments. All the data passed normality verification via the Shapiro–Wilk test, and the homogeneity of variance was confirmed by using the Brown–Forsythe test. The intergroup differences were analyzed using one-way ANOVA, the Bonferroni correction was further applied to adjust the significance level for multiple comparisons, and a *p*-value < 0.05 was considered statistically significant.

## 3. Results

### 3.1. The Novel Hybrid Peptide, VA, Alleviates H_2_O_2_-Induced Cell Damage More Effectively than Its Parental Peptides

**Figure 1:**
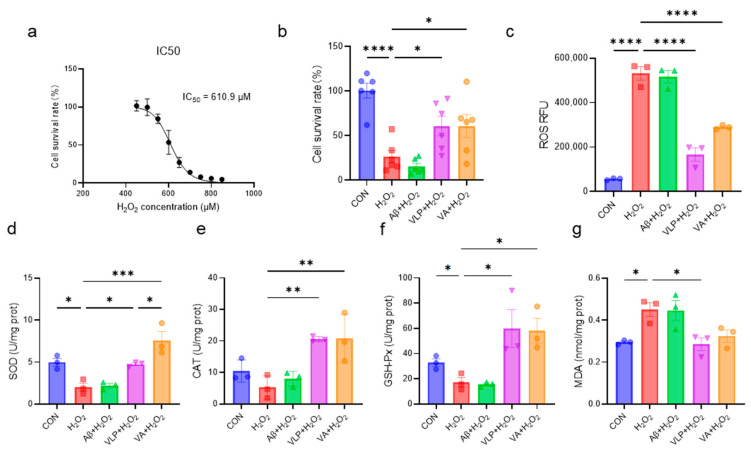
The novel hybrid peptide, VA, alleviates H_2_O_2_-induced cellular damage more effectively than its parental peptides. (**a**) IC50 of H_2_O_2_ on HepG2 cells. Effects of VA, VLP, and Aβ on cell viability (**b**), ROS accumulation (**c**), SOD activity (**d**), CAT activity (**e**), GSH-Px activity (**f**), and MDA levels (**g**) in H_2_O_2_-induced cell damage. The data are presented as the mean ± SEM, and the cell survival rate (**b**) was measured with six independent experimental replicates, while the other assays were performed with three independent experimental replicates. * *p* ≤ 0.05, ** *p* ≤ 0.01, *** *p* ≤ 0.001, **** *p* ≤ 0.0001.

The protective effects of the hybrid peptide, VA, and its parent peptides, Aβ and VLP, against H_2_O_2_-induced HepG2 cell damage were systematically investigated. First, the IC_50_ concentration of H_2_O_2_ was determined as 610.9 μM (Figure 1a). The subsequent evaluation of the protective effects revealed that both VA and VLP significantly improved the cell viability, reduced the intracellular ROS accumulation, and enhanced the antioxidant enzyme activities in HepG2 cells (Figure 1b–f). Notably, the SOD activity in the VA group was significantly higher than that in the VLP group (*p* < 0.05) (Figure 1d). In addition, VA reduced the H_2_O_2_-induced MDA accumulation, although there was no statistically significant difference (Figure 1g). These results indicate that VA alleviates H_2_O_2_-induced oxidative damage in HepG2 cells by enhancing the cellular antioxidant capacity and is more effective than its parent peptide, VLP.

### 3.2. The Novel Hybrid Peptide, VA, Alleviates H_2_O_2_-Induced Cell Damage in a Dose-Dependent Manner

**Figure 2:**
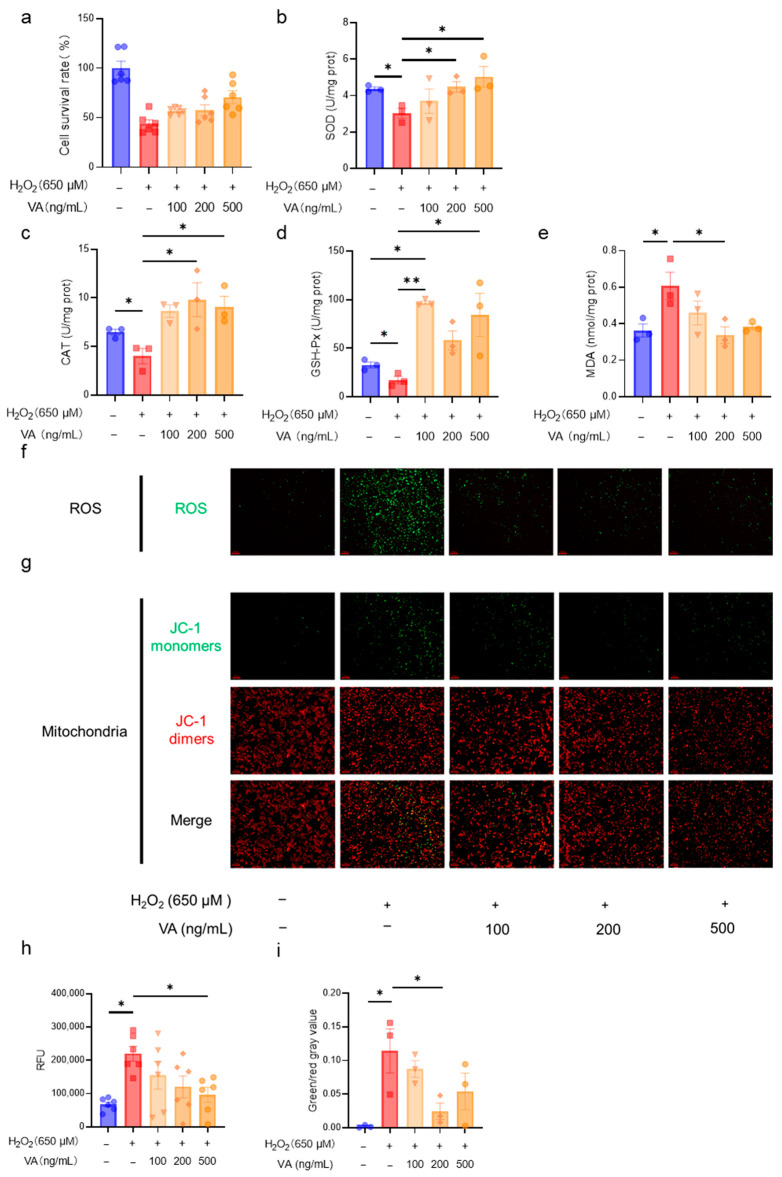
The novel hybrid peptide, VA, alleviates H_2_O_2_-induced cell damage via antioxidant action in a dose-dependent manner. Effect of different concentrations of VA (100, 200, and 500 ng/mL) on cell viability (**a**), SOD activity (**b**), CAT activity (**c**), GSH-Px activity (**d**), and MDA levels (**e**), cellular ROS accumulation (**f**), and mitochondrial damage (**g**) in H_2_O_2_-induced cell damage. (**h**,**i**) are statistical quantitative results for ROS accumulation (**f**) and mitochondrial damage (**g**). The data are presented as the mean ± SEM, and the cell survival rate (**b**) and relative fluorescence intensity of ROS (**h**) were measured with six independent experimental replicates, while the other assays were performed with three independent experimental replicates. * *p* ≤ 0.05, ** *p* ≤ 0.01.

Following the confirmation of VA’s antioxidant activity, further investigations were conducted to assess its dose-dependent effects. HepG2 cells were treated with VA at concentrations of 100, 200, and 500 ng/mL, and the cell viability and antioxidant enzyme activity were assessed. The results demonstrated that the antioxidant activity of VA increased in a concentration-dependent manner (Figure 2a,b,e). Notably, the antioxidant enzyme activities of the H_2_O_2_ + VA group exceeded those of the control group (Figure 2c,d). The intracellular ROS levels were visually analyzed using fluorescence imaging (Figure 2f). H_2_O_2_ treatment markedly increased the green fluorescence, indicating elevated ROS levels, whereas VA pre-treatment reduced ROS accumulation dose-dependently (Figure 2f,h). The mitochondrial integrity was assessed via JC-1 fluorescence microscopy (Figure 2g), and quantitative analysis revealed that H_2_O_2_ increased the proportion of JC-1 monomers, reflecting mitochondrial dysfunction (Figure 2i). Pre-incubation with VA significantly mitigated this effect, thereby preserving the mitochondrial integrity. These results indicate that VA exhibits robust antioxidant activity in a dose dependent manner, effectively reducing the oxidative stress and preserving the mitochondrial function.

### 3.3. The Novel Hybrid Peptide, VA, Alleviates CCl_4_-Induced Liver Damage in Mice by Enhancing the Antioxidant Enzyme Activity

**Figure 3:**
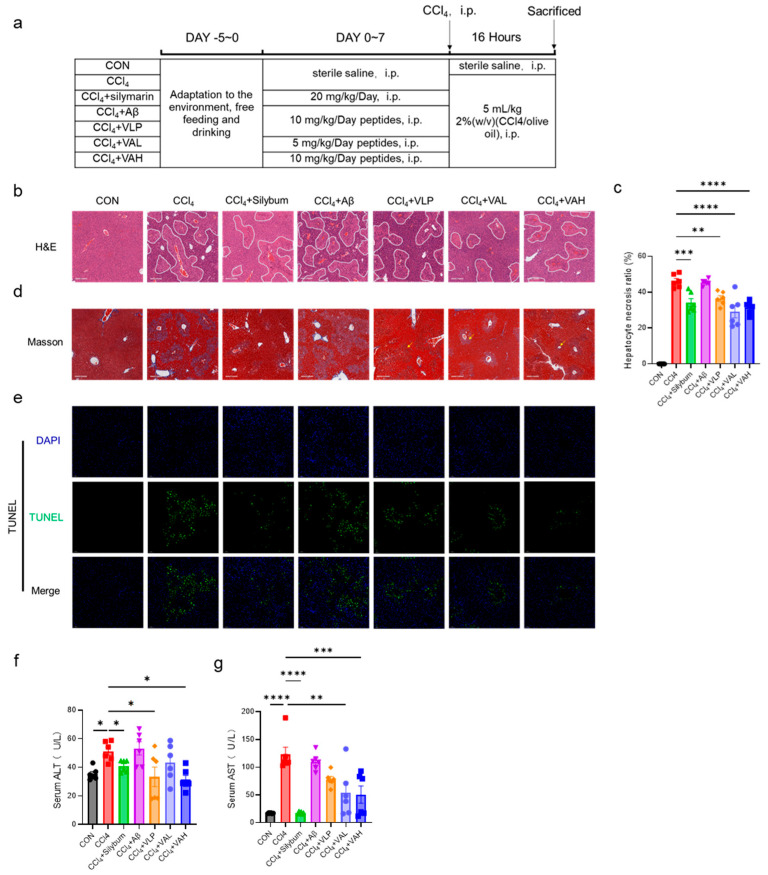
The novel hybrid peptide, VA, alleviates CCl_4_-induced liver damage in mice. (**a**) Experimental protocol for the animal study. (**b**) H&E-stained liver sections and (**d**) Masson-stained liver sections of mice. (**c**) Quantification of necrotic areas in H&E-stained liver sections. (**e**) TUNEL staining of liver tissue. (**f**,**g**) ALT and AST levels in mouse serum. The data are presented as the mean ± SEM (*n* = 6). * *p* ≤ 0.05, ** *p* ≤ 0.01, *** *p* ≤ 0.001, **** *p* ≤ 0.0001.

The in vitro findings were further validated in vivo using an animal model, as cell-based systems have inherent limitations in replicating systemic physiological responses and complex organ-level interactions. To explore the in vivo antioxidant capacity of VA, a carbon tetrachloride (CCl_4_)-induced murine liver injury model was established. Figure 3a illustrates the experimental design, which included seven treatment groups: blank control (CON), CCl_4_ model (CCl_4_), positive control (CCl_4_ + silymarin), parent peptide Aβ group (CCl_4_ + Aβ), parent peptide VLP group (CCl_4_ + VLP), low-dose hybrid peptide VA group (CCl_4_ + VAL), and high-dose hybrid peptide VA group (CCl_4_ + VAH).

The CCl_4_ treatment led to increased liver fibrosis [21], as shown by the H&E staining in Figure 3b, where the fibrotic areas are outlined in white. Silymarin has long been used as a hepatoprotective agent in many parts of the world [22]. It exhibits a strong antioxidant activity, directly scavenging free radicals and reducing their attack on hepatocytes, while also upregulating the activity of intracellular antioxidant enzymes to enhance the liver’s antioxidant defense capacity [23,24]. In this study, as a positive control drug, silymarin demonstrated significant efficacy in alleviating carbon tetrachloride-induced liver injury in mice. Both the low- and high-dose VA treatment significantly reduced the liver tissue damage (Figure 3c). Masson trichrome staining for liver fibrosis further confirmed that VA alleviated liver fibrosis (Figure 3b). Additionally, TUNEL staining was performed to assess the liver cell apoptosis (Figure 3e), and the results showed that VA protected the liver tissue from CCl_4_-induced hepatocyte apoptosis. Notably, both the low- and high-dose VA groups exhibited significantly better anti-apoptotic effects than the parent peptide VLP group (Figure 3e). Consistent with these histological findings, the protective effect of VA was also evidenced by the serum ALT and AST levels (Figure 3f,g). These results collectively indicate that VA possesses potent antioxidant capacity in vivo. Notably, the comparative analysis of the Aβ and VLP treatment groups revealed that VA’s antioxidant activity predominantly originates from its VLP component rather than the Aβ domain.

**Figure 4:**
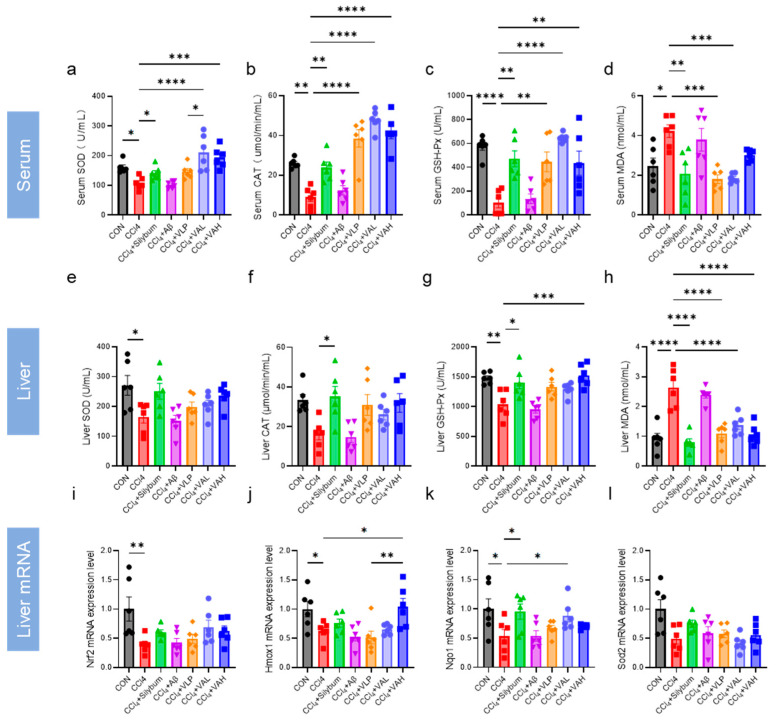
The novel hybrid peptide, VA, enhances antioxidant enzyme activity in mouse liver and serum. (**a**–**d**) Levels of SOD, CAT, GSH-Px, and MDA in mouse serum. (**e**–**h**) Levels of SOD, CAT, GSH-Px, and MDA in mouse liver tissue. (**i**–**l**) mRNA expression levels of genes related to the NRF2-ARE signaling pathway in mouse liver tissue. The data are presented as the mean ± SEM (*n* = 6). * *p* ≤ 0.05, ** *p* ≤ 0.01, *** *p* ≤ 0.001, **** *p* ≤ 0.0001.

The activity of antioxidant enzymes in the serum and hepatic tissues was systematically quantified (Figure 4a–h). The results indicated that VA significantly increased the antioxidant enzyme activity in both the serum and liver, thereby enhancing the overall antioxidant capacity in mice. Moreover, the VA treatment led to significantly higher SOD in the serum compared to the VLP group, indicating that VA exhibited stronger antioxidant capacity in vivo than its parent peptide, VLP.

The expression levels of the key genes in the antioxidant regulatory pathway (*Nrf2*, *Hmox1*, *Nqo1*, and *Sod2*) were further quantified (Figure 4i–l). The results revealed that VA enhanced the expression of *Nrf2*, *Hmox1*, and *Nqo1*, and this effect was superior to that of the parent peptide, VLP (Figure 4j).

### 3.4. The Antioxidant Activity of the Hybrid Peptide, VA, Is Dependent on NRF2

**Figure 5:**
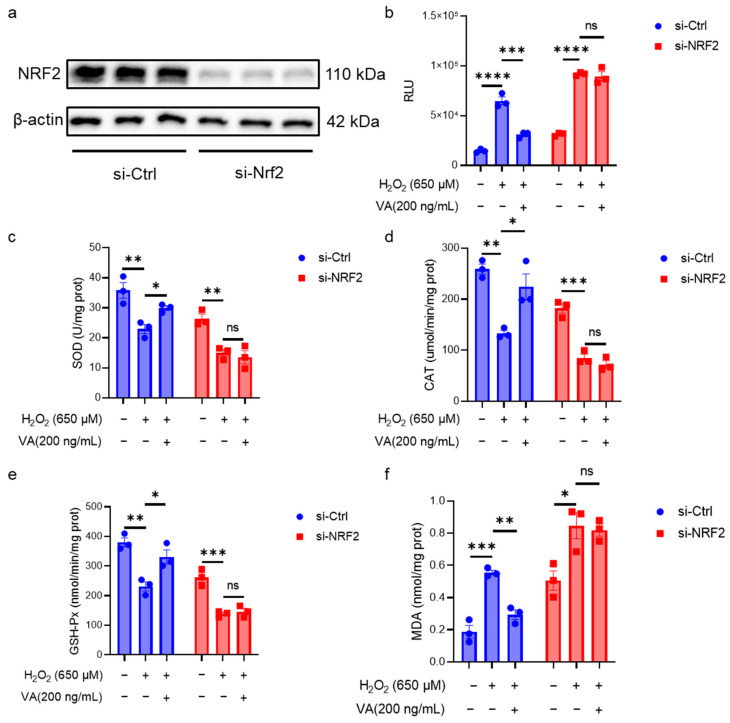
The antioxidant activity of hybrid peptide, VA, depends on the NRF2. (**a**) Western blot analysis of NRF2 after siRNA-mediated NRF2 knockdown in H_2_O_2_-induced HepG2 cell damage. (**b**) Measurement of ROS accumulation levels after siRNA-mediated NRF2 knockdown in H_2_O_2_-induced HepG2 cell damage. (**c**–**f**) Measurement of SOD, GSH-Px, CAT, and MDA levels after siRNA-mediated NRF2 knockdown in H_2_O_2_-induced HepG2 cell damage. The data are presented as the mean ± SEM (*n* = 3). ^ns^ *p* ≥ 0.05, * *p* ≤ 0.05, ** *p* ≤ 0.01, *** *p* ≤ 0.001, **** *p* ≤ 0.0001.

NRF2 is a crucial transcription factor in the body’s antioxidant defense system. Although the previous data in this study suggested that VA has an effect on the NRF2 expression, it is not clear whether its antioxidant activity is dependent on NRF2. To overcome the inherent limitations of animal models in mechanistic studies due to the organismal complexity, this investigation employed HepG2 cell models for the in-depth exploration of VA’s antioxidant mechanisms. To further elucidate the role of NRF2 in VA-mediated antioxidant effects, NRF2 was knocked down in HepG2 cells using siRNA (Figure 5). Western blot analysis confirmed that the NRF2 knockdown cell line was successfully constructed (Figure 5a). The intracellular antioxidant enzyme activity and ROS levels in NRF2 knockdown cells were measured (Figure 5b–f). As expected, NRF2 knockdown resulted in the loss of VA’s antioxidant activity. These results confirm that the antioxidant activity of VA is dependent on NRF2.

### 3.5. The Novel Hybrid Peptide, VA, Alleviates Oxidative Stress by Modulating the KEAP1-NRF2-ARE Signaling Pathway

**Figure 6:**
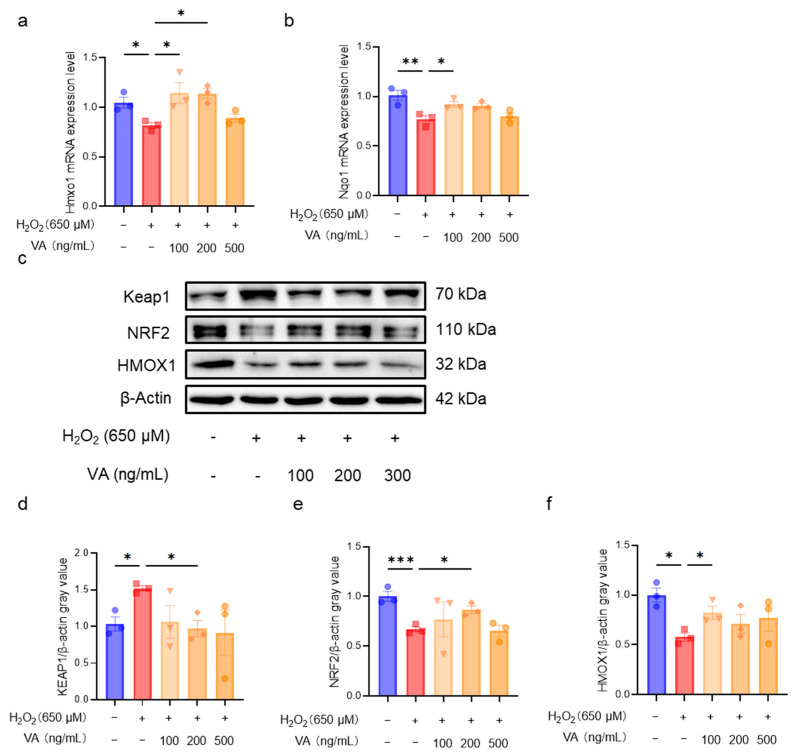
The novel hybrid peptide, VA, alleviates oxidative stress by modulating the KEAP1-NRF2-ARE signaling pathway. VA enhances NRF2 expression by reducing mRNA expression levels of genes related to the NRF2-ARE signaling pathway in H_2_O_2_-induced HepG2 cell damage (**a**,**b**). (**c**) Western blot analysis of KEAP1, NRF2, and HMOX1 expression levels in H_2_O_2_-induced HepG2 cell damage. (**d**–**f**) Relative quantification of Western blot results for KEAP1, NRF2, and HMOX1. The data are presented as the mean ± SEM (*n* = 3). * *p* ≤ 0.05, ** *p* ≤ 0.01, *** *p* ≤ 0.001.

Through in vitro and in vivo experiments, the excellent antioxidant activity of VA was confirmed to be dependent on the NRF2-ARE pathway. To further investigate the mechanism by which VA regulates this pathway, the expression levels of the key genes (*Hmox1*, *Nqo1*) in the KEAP1-NRF2-ARE pathway were first measured in cells (Figure 6a,b). H_2_O_2_ treatment downregulated the expression levels of *Hmox1* and *Nqo1*, whereas pre-incubation with VA significantly upregulated their expression (*p* < 0.05), consistent with the results observed in the mouse experiments. Next, Western blot analysis was performed to evaluate the expression levels of the key proteins in the KEAP1-NRF2-ARE pathway (Figure 6c). The H_2_O_2_ treatment group exhibited a reduced NRF2 expression, which subsequently affected the expression of the NRF2-regulated protein, HMOX1. As expected, pre-incubation with VA significantly restored the expression of NRF2 (Figure 6e) and HMOX1 (Figure 6f). VA upregulates NRF2 expression primarily by downregulating KEAP1 expression (Figure 6d).

## 4. Discussion

ROS can disrupt the redox balance in the body and can further cause various diseases [1]. Antioxidants play a crucial role in scavenging excess free radicals and restoring cellular homeostasis [25,26]. Among the various antioxidant agents, peptides have attracted great attention in agricultural, food, and clinical fields due to their low toxicity, high efficacy, and easy absorption [27,28,29,30]. Consequently, antioxidant peptides have great potential for addressing oxidative stress and its associated diseases. However, there are still some challenges remaining, such as the low efficiency in identifying or developing effective antioxidant peptides.

To address this issue, a previous study established a high throughput screening platform for multifunctional peptides, and successfully identified a hybrid peptide, VA, with both immunomodulatory and antioxidant activities [13]. This study aimed to comprehensively evaluate the antioxidant activity of VA and elucidate its underlying molecular mechanisms, with a focus on its potential applications in mitigating oxidative stress-related diseases.

Under cellular oxidative stress conditions, excessive reactive oxygen species (ROS) are generated, with hydrogen peroxide (H_2_O_2_) being one of the predominant ROS types. An in vitro H_2_O_2_-induced cell model can effectively simulate the oxidative stress state observed in vivo. Therefore, an H_2_O_2_-induced oxidative damage cell model is commonly used in in vitro antioxidants research. H_2_O_2_ can cross the cell membrane and generate hydroxyl and oxygen radicals, leading to cellular oxidative stress [31]. HepG2 cells, derived from a human hepatocellular carcinoma cell line, retain multiple metabolic functions characteristic of hepatocytes and possess intact redox metabolic pathways. Consequently, they are widely utilized as an in vitro cell model for studying oxidative stress and metabolic processes [32]. This study constructed an H_2_O_2_-induced HepG2 cell oxidative damage model to investigate the antioxidant properties of VA (Figure 1). Additionally, in vitro studies have limitations, and individual cell lines sometimes cannot fully reflect the complete effects of antioxidants, so in vivo studies are essential. Therefore, a CCl_4_-induced mouse liver damage model was employed for the in vivo investigation of VA. When CCl_4_ enters hepatocytes, it is metabolized by the cytochrome P450 enzyme family (CYP family) to produce the trichloromethyl radical (CCl_3_•) and trichloromethyl peroxide radical (Cl_3_COO•) [33]. These free radicals are highly reactive and unstable, attacking proteins and lipids, and weakening the antioxidant defense by depleting reductants and inhibiting antioxidant enzyme production [33]. This pathological cascade effectively recapitulates the oxidative stress processes observed in hepatic tissues. A typical phenotype of CCl_4_ exposure is liver fibrosis [21], as observed in this work (Figure 3b,d). The antioxidant capacity of VA was systematically investigated through integrated in vitro and in vivo approaches.

In the H_2_O_2_-induced HepG2 model, oxidative stress leads to ROS accumulation, mitochondrial damage, and ultimately cell death (Figure 2). However, VA effectively prevents H_2_O_2_-induced damage. Similarly, in the CCl_4_-induced liver damage model, VA exhibited excellent antioxidant capacity, protecting hepatocytes from CCl_4_-induced fibrosis (Figure 3b,d) and reducing hepatocyte apoptosis (Figure 3e). Moreover, VA’s antioxidant capacity increased in a dose-dependent manner. Remarkably, VA exhibited a stronger antioxidant activity compared to its parent peptide, VLP. This may be related to changes in the amino acid composition, sequence length, and spatial structure of the peptide [34,35]. This is an area that deserves further exploration in the future studies.

The KEAP1-NRF2-ARE pathway plays an important role in the body’s antioxidant defense system [36]. ROS and other endogenous or exogenous stimuli activate NRF2, which promotes the expression of downstream antioxidant enzymes, including SOD, CAT, and GSH-Px [37,38,39,40]. These antioxidant enzymes then scavenge the stressors and restore the redox balance. Thus, the activity levels of antioxidant enzymes reflect the efficiency of antioxidants [10]. Both in vitro (Figure 2) or in vivo (Figure 4), the hybrid peptide, VA, could increase the activity of antioxidant enzymes reduced by oxidative damage. Furthermore, as the end product of lipid peroxidation, MDA increases when excessive ROS generation exceeds the scavenging capacity of antioxidant enzymes such as SOD, CAT, and GSH-Px, thereby triggering lipid peroxidation [41]. The measurement of the MDA content in this study exhibited the same trend: MDA levels rose when antioxidant enzyme activity was low, while VA supplementation enhanced the antioxidant enzyme activity and subsequently reduced the MDA content (Figure 2e and Figure 4d,h). Further investigation revealed that the antioxidant activity of VA was almost completely eliminated upon the knockdown of NRF2 expression (Figure 5). This also indicates that VA regulates the NRF2-ARE signaling pathway to exert its antioxidant activity. Furthermore, VA not only significantly restored the activity of cellular antioxidant enzymes after H_2_O_2_ damage, but even enabled it to surpass that of the blank control group (Figure 2b–d). Its antioxidant capacity is superior to most of the previously reported peptides, demonstrating that VA has exceptional antioxidant capacity [11,42,43].

Under normal conditions, NRF2 forms a complex with KEAP1 in the cytoplasm, where it is predominantly degraded via ubiquitination [36]. However, during oxidative stress, the KEAP1-NRF2 complex dissociates, resulting in a prolonged half-life of NRF2 and subsequent translocation into the nucleus. In the nucleus, NRF2 binds to antioxidant response elements (AREs) and activates the expression of various downstream antioxidant genes [11,44]. The Western blot results confirm this (Figure 6c). VA enhanced NRF2 protein expression and further upregulated the downstream regulatory protein, HMOX1. Notably, VA promoted NRF2 expression by reducing the KEAP1 levels. There are several pathways that can reduce the KEAP1 expression, such as chaperone-mediated autophagy, which directly binds to KEAP1 and induces its ubiquitin-mediated degradation [45]. Some peptides can also directly bind to KEAP1 to promote its degradation [11,46]. However, the specific mechanism by which VA reduces the KEAP1 expression remains unclear and warrants further investigation.

## 5. Conclusions

In conclusion, our study demonstrated that the hybrid peptide, VA, exhibits strong antioxidant activity both in vitro and in vivo. In vitro, VA can alleviate the oxidative damage of HepG2 cells induced by H_2_O_2_, increase the activity levels of the antioxidant enzymes (SOD, CAT, and GSH-Px) within the cells, and reduce the accumulation of MDA. At the same time, it alleviates the level of the mitochondrial damage within the cells and reduces the accumulation of reactive oxygen species (ROS). In vivo, VA can alleviate the oxidative damage of the liver in mice induced by carbon tetrachloride. It can increase the activity levels of the antioxidant enzymes (SOD, CAT, and GSH-Px) in both the serum and the liver, reduce the accumulation level of MDA, alleviate liver fibrosis, and decrease the apoptosis level of liver cells. The antioxidant activity of VA is dependent on NRF2. Further studies revealed that VA enhanced the antioxidant capacity by activating the KEAP1-NRF2-ARE signaling pathway; specifically, VA blocked the KEAP1-mediated degradation of NRF2 and thus promoted the expression of its downstream antioxidant enzymes. These findings provide strong theoretical guidance and technical support for the use of VA as a potential antioxidant for agricultural, food, and clinical applications, as well as for its industrial development.

## Figures and Tables

**Table 1:** Sequence information of peptides.

Peptide	Sequence
Aβ	DAEFRHDSGYEVHHQKLVFFAEDVGSNKGAIIGLMVG
VLP	VLPVPQK
VLP-Aβ	VLPVPQKDAEFRHDSGYEVHHQKLVFFAEDVGSNKGAIIGLMVG

**Table 2:** Sequences of the primers used for RT-PCR assays.

Gene Name	Gene Accession Numbers		Sequence (5′–3′)	Length
*mNrf2*	NM_010902.5	F	GCTGGCTGATACTACCGCTGTTC	23
		R	AGTGGAGAGGATGCTGCTGAAAGA	24
*mHmox1*	NM_010442.2	F	AGACCGCCTTCCTGCTCAACA	21
		R	CTCTGACGAAGTGACGCCATCTG	23
*mNqo1*	NM_008706.5	F	GCGAGAAGAGCCCTGATTGTACTG	24
		R	GCCTCTACAGCAGCCTCCTTCA	22
*mSod2*	NM_013671.3	F	TCCCAGACCTGCCTTACGACTATG	24
		R	TTGATAGCCTCCAGCAACTCTCCTT	25
*mβ-actin*	NM_007393.5	F	TCACTATTGGCAACGAGCGGTTC	23
		R	CAGCACTGTGTTGGCATAGAGGTC	24
*hHmox1*	NM_002133.3	F	GCCAGCAACAAAGTGCAAGA	20
		R	TAAGGACCCATCGGAGAAGC	20
*hNqo1*	NM_000903.3	F	TGGTGGAGTCGGACCTCTATG	21
		R	CATGGCAGCGTAAGTGTAAGC	21
*hβ-actin*	NM_001101.5	F	ATCGTCCACCGCAAATGCTTCT	22
		R	TGCTGTCACCTTCACCGTTCCA	22

## Data Availability

Data are contained within the article.
